# Community antibiotic self-medication, pharmacy access, and WHO AWaRe classification of self-medicated antibiotics among adult antibiotic users in Narayanganj Sadar, Bangladesh: a multistage stratified cluster-sampled cross-sectional study

**DOI:** 10.1016/j.ijregi.2026.101009

**Published:** 2026-09-11

**Authors:** Syed Amir Al Shahid Rhode, Tairin Sultana, Mahzabin, Aaqib Adnan Likhon, Alif Ahnaf Shikhon, Rifa Rafia

**Affiliations:** 1 Bangladesh Health Research & Implementation Initiative (BHRI), Narayanganj, Bangladesh; 2 Department of Applied Health and Nutrition, RTM Al-Kabir Technical University, Sylhet, Bangladesh; 3 Shaheed Suhrawardy Medical College & Hospital, Dhaka, Bangladesh; 4 Bangladesh Medical University (BMU), Dhaka, Bangladesh; 5 Continental Hospital PLC, Dhaka, Bangladesh

**Keywords:** Antibiotic self-medication, Community pharmacy, Antimicrobial stewardship, Antimicrobial resistance, WHO AWaRe classification, Bangladesh

## Abstract

**Objectives:**

To estimate the 6-month proportion of antibiotic self-medication among adults with recent antibiotic use in Narayanganj Sadar, Bangladesh, and examine associated characteristics.

**Methods:**

This community-based cross-sectional study enrolled 400 adults from 50 clusters across four sampling strata between January and April 2026. The most recent antibiotic course within the preceding 6 months was the index course. Self-medication was defined by the absence of prescribing involvement from a Bangladesh Medical and Dental Council-registered physician. Design-aware modified Poisson regression estimated adjusted prevalence ratios, accounting for clustering and stratification. Self-medicated antibiotics were classified using the World Health Organization Access, Watch, and Reserve framework.

**Results:**

Self-medication occurred in 244/400 participants (61.0%; 95% confidence interval 55.6%-66.4%). Higher prevalence was associated with pharmacy access (adjusted prevalence ratio 1.50, 95% confidence interval 1.29-1.74), poor antibiotic knowledge (1.46, 1.24-1.73), lower educational attainment (1.30, 1.09-1.55), and lack of antimicrobial-resistance awareness (1.22, 1.06-1.41). Watch or Reserve agents accounted for 145/201 classifiable episodes (72.1%) and 117/152 objectively verified episodes (77.0%).

**Conclusion:**

Antibiotic self-medication was common among recent antibiotic users. Pharmacy access and limited antimicrobial literacy were associated characteristics rather than demonstrated causal determinants. Higher-priority AWaRe exposure remained substantial.

## Highlights

• Antibiotic self-medication occurred in 61% of recent antibiotic users.
• Pharmacy access was associated with higher self-medication prevalence.
• Poor knowledge and lower education were associated with self-medication.
• Watch or Reserve agents comprised 72% of classifiable self-medication episodes.
• Design-aware analyses accounted for 50 clusters across four sampling strata.

## Introduction

Antimicrobial resistance (AMR) is a major global health threat. In 2019, bacterial AMR was associated with an estimated 4.95 million deaths worldwide, including 1.27 million deaths directly attributable to resistance [1]. Antibiotic self-medication and other forms of non-prescription use can expose individuals to antimicrobial treatment without appropriate clinical assessment and may contribute to unnecessary antibiotic exposure, inappropriate agent selection, delayed care, and selection pressure for resistance. Such practices remain common in low- and middle-income countries, where healthcare access, medicine availability, prior treatment experience, cost, and informal care pathways can influence decisions about antibiotic use [2,3].

In Bangladesh, antimicrobial governance and enforcement remain challenging [4], making the distinction between who directs antibiotic use and where the antibiotic is obtained particularly important. Community pharmacies and drug shops can supply medicines prescribed within the formal health system while also providing a route to antibiotics obtained without a registered physician’s prescription. Previous studies illustrate the scale and heterogeneity of these practices. A community survey in Rajshahi City reported antibiotic self-medication among 26.7% of participants [5]. More recent pharmacy-based research found that 56.6% of antibiotic purchases occurred without a prescription and that 73.5% of purchased antibiotics belonged to the World Health Organization (WHO) Watch group [6]. In another pharmacy survey spanning urban and rural sites across Bangladesh, approximately half of antibiotic courses were purchased without a registered physician’s prescription; Watch and Reserve antibiotics accounted for 53.6% and 10.0% of dispensed courses, respectively [7]. The WHO Access, Watch, and Reserve (AWaRe) framework therefore provides a useful standardized approach for characterizing the stewardship profile of antibiotics used in the community [8].

Existing evidence from Bangladesh, however, has addressed different components of community antibiotic use in distinct populations and settings. Community studies have estimated self-medication and examined associated sociodemographic and knowledge-related characteristics [5,9]; university-based studies have focused on antibiotic knowledge, practices, and self-medication [10]; and pharmacy-based studies have characterized non-prescription purchasing, dispensing pathways, and AWaRe composition [6,7]. These studies provide complementary evidence, but, to our knowledge, they have not been integrated within a community-based sample of recent adult antibiotic users, the prescribing status of a common index course, its acquisition source, antibiotic knowledge, AMR awareness, reported indication, and antibiotic-level AWaRe classification. This distinction is methodologically important because acquisition from a pharmacy does not necessarily imply non-prescription use: pharmacies may supply antibiotics prescribed by qualified clinicians as well as antibiotics obtained through other pathways.

Narayanganj Sadar provides a strategically informative setting in which to examine these dimensions together. Industrial urban communities coexist within a geographically compact area alongside semi-urban, peri-urban, and rural settlements with differing socioeconomic and healthcare-access environments. The setting was selected to capture this internal heterogeneity rather than to serve as a proxy for Bangladesh as a whole. The primary objective of this study was therefore to estimate the 6-month proportion of antibiotic self-medication among adults with recent antibiotic use in Narayanganj Sadar and to examine associated sociodemographic, antibiotic-knowledge, AMR-awareness, and pharmacy-access characteristics. Secondary objectives were to describe antibiotic acquisition sources and reported indications and to classify identifiable self-medicated antibiotics according to the WHO AWaRe framework.

## Methods

### Study design and participants

We conducted a community-based cross-sectional study in Narayanganj Sadar, Bangladesh, from January to April 2026. Adults aged ≥18 years were eligible if they had lived in the study area for at least 6 months and had used an antibiotic during the preceding 6 months. Healthcare professionals and healthcare students were excluded. The study was designed as a subnational investigation and was not intended to provide nationally representative estimates. Reporting followed Strengthening the Reporting of Observational Studies in Epidemiology guidance [11].

When participants reported more than one antibiotic course during the 6-month recall period, the most recent course was defined as the index course. All antibiotic-specific variables, including prescription status, acquisition source, indication, and antibiotic identity, referred to this same episode.

### Sampling and data collection

A multistage stratified cluster-sampling design was used. Narayanganj Sadar was stratified into urban, semi-urban, peri-urban, and rural areas, from which 50 operational clusters were selected. Eight participants were recruited from each cluster, yielding 400 participants. Households were selected systematically after a random start, and when more than one eligible adult was present, one was selected by lottery. Detailed sampling procedures are provided in the Supplementary Methods. The original study-size calculation assumed a 50% proportion, 95% confidence level, and 5% absolute margin of error, yielding 384 participants; the target was increased to 400. The calculation did not include a design-effect adjustment for clustering.

Data were collected through face-to-face interviews using a structured questionnaire developed for the study, reviewed by a multidisciplinary expert group, translated into Bangla, pilot tested before field implementation, and administered by trained data collectors. The instrument was not a formally validated psychometric questionnaire.

### Outcome and study variables

The primary outcome was antibiotic self-medication for the index course. A physician was operationally defined as an Bachelor of Medicine, Bachelor of Surgery (MBBS) or Bachelor of Dental Surgery (BDS) doctor registered with the Bangladesh Medical and Dental Council. Antibiotic use without prescribing involvement from such a physician was classified as self-medication; use involving physician prescribing was classified as physician-prescribed.

Antibiotic acquisition source was recorded separately as pharmacy, previous prescription, family/friend, online source, leftover medicine, or other. Pharmacy access was defined as reporting a pharmacy as the acquisition source for the index course. It was treated as an access characteristic rather than as evidence of pharmacy recommendation or a causal exposure. Prescription status and acquisition source were audited against the original field records during revision; details are provided in the Supplementary Methods.

Covariates included age, sex, marital status, educational attainment, monthly household income, household size, antibiotic knowledge, and awareness of AMR. Poor antibiotic knowledge was a single-item operational indicator based on bacterial-versus-viral recognition and was not a validated knowledge scale. Knowledge and AMR awareness were measured at interview; their temporal ordering relative to the index antibiotic course could not be established.

### Antibiotic identity and WHO AWaRe classification

Among self-medication episodes, antibiotic identity was established from the best available supporting information, including medicine strips, packages, or containers, photographs, or participant report. Identifiable antibiotics were classified according to the 2025 WHO AWaRe framework [8]. Episodes for which antibiotic identity could not be established were retained as unclassifiable. In addition to the primary AWaRe distribution among classifiable episodes, sensitivity analyses examined all self-medication episodes, extreme assumptions for unclassifiable episodes, and episodes with objectively verified antibiotic identities. The verification hierarchy and sensitivity definitions are described in the Supplementary Methods.

### Statistical analysis

Participant characteristics were summarized using frequencies and percentages. Unadjusted comparisons in Tables 1, 2, and 4 used global design-aware Wald tests from crude modified Poisson models. The reconstructed sampling design specified 50 primary sampling units across four strata. Because the original information required to reconstruct exact sampling weights was not fully retained, analyses were unweighted.

**Table 1:** Participant characteristics and sampling-stratum distribution according to antibiotic self-medication status.

Characteristic	Overall, n (%)	Self-medication, n/N (%)	Physician-prescribed, n/N (%)	*P*-value
**Sampling stratum**				**—**
Urban	104 (26.0)	64/104 (61.5)	40/104 (38.5)	
Semi-urban	96 (24.0)	56/96 (58.3)	40/96 (41.7)	
Peri-urban	96 (24.0)	60/96 (62.5)	36/96 (37.5)	
Rural	104 (26.0)	64/104 (61.5)	40/104 (38.5)	
**Age, years**				**0.744**
18-29	88 (22.0)	57/88 (64.8)	31/88 (35.2)	
30-39	121 (30.3)	72/121 (59.5)	49/121 (40.5)	
40-49	103 (25.8)	59/103 (57.3)	44/103 (42.7)	
50-59	53 (13.3)	32/53 (60.4)	21/53 (39.6)	
≥60	35 (8.8)	24/35 (68.6)	11/35 (31.4)	
**Sex**				**0.299**
Male	232 (58.0)	147/232 (63.4)	85/232 (36.6)	
Female	168 (42.0)	97/168 (57.7)	71/168 (42.3)	
**Educational attainment**				**0.008**
No formal education	103 (25.8)	75/103 (72.8)	28/103 (27.2)	
Secondary	25 (6.3)	20/25 (80.0)	5/25 (20.0)	
Higher secondary	43 (10.8)	28/43 (65.1)	15/43 (34.9)	
Graduate	157 (39.3)	86/157 (54.8)	71/157 (45.2)	
Postgraduate	72 (18.0)	35/72 (48.6)	37/72 (51.4)	
**Monthly household income**				**0.018**
<30,000 BDT	117 (29.3)	83/117 (70.9)	34/117 (29.1)	
30,000-60,000 BDT	141 (35.3)	78/141 (55.3)	63/141 (44.7)	
>60,000 BDT	142 (35.5)	83/142 (58.5)	59/142 (41.5)	
**Marital status**				**0.549**
Married	244 (61.0)	145/244 (59.4)	99/244 (40.6)	
Unmarried	70 (17.5)	45/70 (64.3)	25/70 (35.7)	
Widowed	51 (12.8)	34/51 (66.7)	17/51 (33.3)	
Divorced	35 (8.8)	20/35 (57.1)	15/35 (42.9)	
**Household members**				**0.082**
2	30 (7.5)	14/30 (46.7)	16/30 (53.3)	
3	72 (18.0)	39/72 (54.2)	33/72 (45.8)	
4	271 (67.8)	174/271 (64.2)	97/271 (35.8)	
≥5	27 (6.8)	17/27 (63.0)	10/27 (37.0)	

Data are *n* (%) unless otherwise indicated. The overall 6-month proportion of antibiotic self-medication among recent antibiotic users was 244/400 (61.0%; design-aware 95% CI 55.6%-66.4%). *P*-values account for the reconstructed sampling design (50 primary sampling units across four strata) and are unweighted. Sampling stratum is shown descriptively and was not tested as an ordinary participant-level covariate because it formed part of the sampling design.

BDT, Bangladeshi taka; CI, confidence interval.

**Table 2:** Antibiotic knowledge, antimicrobial-resistance awareness, and pharmacy access according to self-medication status.

Characteristic	Overall, n (%)	Self-medication, n/N (%)	Physician-prescribed, n/N (%)	*P*-value
**Antibiotic knowledge**				**<0.001**
Adequate	210 (52.5)	102/210 (48.6)	108/210 (51.4)	
Poor	190 (47.5)	142/190 (74.7)	48/190 (25.3)	
**Awareness of antimicrobial resistance**				**0.003**
Aware	280 (70.0)	158/280 (56.4)	122/280 (43.6)	
Not aware	120 (30.0)	86/120 (71.7)	34/120 (28.3)	
**Pharmacy access**				**<0.001**
No	200 (50.0)	93/200 (46.5)	107/200 (53.5)	
Yes	200 (50.0)	151/200 (75.5)	49/200 (24.5)	
**Belief that antibiotics treat viral illness**				**<0.001**
No	278 (69.5)	155/278 (55.8)	123/278 (44.2)	
Yes	122 (30.5)	89/122 (73.0)	33/122 (27.0)	

Pharmacy access was defined as reporting a pharmacy as the acquisition source for the index antibiotic course. No separate pharmacy-recommendation construct is inferred from the analytical dataset. “Poor antibiotic knowledge” is a single-item operational indicator based on bacterial-versus-viral recognition and is not a validated multi-item knowledge scale. Belief that antibiotics treat viral illness is a component of this indicator and was not entered separately in the multivariable model. *P*-values account for the reconstructed cluster/stratum design and are unweighted.

The proportion of self-medication among recent antibiotic users and its 95% confidence interval (CI) were estimated using design-aware variance estimation accounting for clustering and stratification. Associations were examined using modified Poisson regression with a log link to estimate crude and adjusted prevalence ratios (aPRs) with 95% CIs [12]. The adjusted model included age, sex, marital status, education, household income, household size, antibiotic knowledge, AMR awareness, and pharmacy access. Household size was modeled as 2, 3, 4, and 5 members. Inference used the reconstructed stratified-cluster design with 46 design degrees of freedom.

Logistic regression using the same covariates and sampling design was performed as a sensitivity analysis; modified Poisson PRs remained the primary measures because self-medication was common. Tests were two-sided, with *P* < 0.05 considered statistically significant. Further details of sampling reconstruction, variable provenance, audit procedures, and design-aware variance estimation are provided in the Supplementary Methods.

### Ethical considerations

Ethical approval was obtained from the Department of Applied Health and Nutrition, RTM Al-Kabir Technical University, Bangladesh, on 10 December 2025 (approval no. 0992420007273006). All participants provided informed consent before enrollment.

## Results

### Participant characteristics and antibiotic self-medication

All 400 enrolled participants were included in the analysis; counts of individuals screened, found ineligible, or declining participation were not retained. Participants were recruited from 50 clusters across the four sampling strata: 104 (26.0%) from urban, 96 (24.0%) from semi-urban, 96 (24.0%) from peri-urban, and 104 (26.0%) from rural areas. The largest age group was 30-39 years (121/400, 30.3%), 232 (58.0%) participants were male, and 229 (57.3%) had graduate or postgraduate education (Table 1).

Antibiotic self-medication was identified in 244/400 participants, corresponding to a 6-month proportion of 61.0% (design-aware 95% CI 55.6-66.4%) among recent antibiotic users. The remaining 156 participants (39.0%) had physician-prescribed index courses. Self-medication differed across educational-attainment categories (*P* = 0.008) and household-income categories (*P* = 0.018), but not significantly by age, sex, marital status, or household size (Table 1).

### Antibiotic knowledge, AMR awareness, and pharmacy access

Poor antibiotic knowledge was identified in 190 participants (47.5%), 120 (30.0%) had not heard of antimicrobial resistance, and 200 (50.0%) reported a pharmacy as the acquisition source for the index antibiotic course. Self-medication occurred in 74.7% of participants with poor antibiotic knowledge compared with 48.6% of those with adequate knowledge; in 71.7% of those without AMR awareness compared with 56.4% of those who were aware; and in 75.5% of participants with pharmacy access compared with 46.5% of those without pharmacy access. Each of these associations remained significant in design-aware comparisons (Table 2).

### Factors associated with antibiotic self-medication

In the design-aware modified Poisson model, pharmacy access was associated with a higher prevalence of self-medication after adjustment for the prespecified covariates (aPR 1.50, 95% CI 1.29-1.74; *P* < 0.001). Poor antibiotic knowledge was similarly associated with self-medication (aPR 1.46, 95% CI 1.24-1.73; *P* < 0.001), as were lower educational attainment (aPR 1.30, 95% CI 1.09-1.55; *P* = 0.005) and absence of AMR awareness (aPR 1.22, 95% CI 1.06-1.41; *P* = 0.008) (Table 3).

**Table 3:** Design-aware modified Poisson regression of factors associated with antibiotic self-medication.

Predictor	Crude PR (95% CI)	Crude *P*-value	Adjusted PR (95% CI)	Adjusted *P*-value
**Age 30-39 vs 18-29 years**	0.92 (0.72-1.17)	0.484	1.07 (0.85-1.34)	0.572
**Age 40-49 vs 18-29 years**	0.88 (0.70-1.11)	0.289	1.04 (0.84-1.27)	0.728
**Age 50-59 vs 18-29 years**	0.93 (0.71-1.23)	0.615	1.15 (0.87-1.52)	0.311
**Age ≥60 vs 18-29 years**	1.06 (0.78-1.44)	0.708	1.08 (0.82-1.42)	0.584
**Female vs male**	0.91 (0.76-1.09)	0.299	0.90 (0.75-1.07)	0.237
**Unmarried vs married**	1.08 (0.89-1.32)	0.430	1.11 (0.92-1.34)	0.264
**Widowed vs married**	1.12 (0.94-1.34)	0.205	1.07 (0.88-1.31)	0.486
**Divorced vs married**	0.96 (0.71-1.30)	0.795	0.98 (0.75-1.27)	0.865
**Low education vs graduate/postgraduate**	1.36 (1.15-1.61)	<0.001	1.30 (1.09-1.55)	0.005
**Monthly income <30,000 vs 30,000 BDT**	1.25 (1.07-1.45)	0.005	1.13 (0.97-1.32)	0.121
**Household size 3 vs 2**	1.16 (0.85-1.59)	0.344	1.02 (0.74-1.41)	0.882
**Household size 4 vs 2**	1.38 (1.06-1.79)	0.019	1.14 (0.88-1.47)	0.303
**Household size ≥5 vs 2**	1.35 (0.92-1.98)	0.121	1.10 (0.78-1.54)	0.586
**Poor antibiotic knowledge**	1.54 (1.30-1.82)	<0.001	1.46 (1.24-1.73)	<0.001
**No antimicrobial-resistance awareness**	1.27 (1.09-1.48)	0.003	1.22 (1.06-1.41)	0.008
**Pharmacy access**	1.62 (1.40-1.89)	<0.001	1.50 (1.29-1.74)	<0.001

Estimates were obtained using modified Poisson regression with a log link and an unweighted stratified-cluster sandwich variance estimator specifying 50 reconstructed primary sampling units across four sampling strata; inference used 46 design degrees of freedom. The adjusted model included all predictors shown. Reference groups were age 18-29 years, male sex, married status, graduate/postgraduate education, income ≥30,000 BDT, household size 2, adequate antibiotic knowledge, antimicrobial-resistance awareness, and no pharmacy access. Low education comprised no formal, secondary, or higher-secondary education. Global adjusted Wald tests were: age group F(4, 46) = 0.348, *P* = 0.844; marital status F(3, 46) = 0.542, *P* = 0.656; household size F(3, 46) = 0.695, *P* = 0.560. Pharmacy access denotes pharmacy as the reported acquisition source and is interpreted as an association, not a causal effect.

BDT, Bangladeshi taka; CI, confidence interval; PR, prevalence ratio.

The crude association between monthly household income <30,000 Bangladeshi taka and self-medication was attenuated after adjustment and was not statistically significant (aPR 1.13, 95% CI 0.97-1.32; *P* = 0.121). Age, sex, marital status, and household size were also not independently associated with self-medication in the adjusted model. Global adjusted tests were non-significant for age group (*P* = 0.844), marital status (*P* = 0.656), and household size (*P* = 0.560).

The design-aware logistic sensitivity analysis produced associations in the same direction as the primary modified Poisson model, although the corresponding odds ratios were farther from the null, as expected for a common outcome (Supplementary Tables S1 and S2).

### Antibiotic acquisition sources

Pharmacies were the most frequently reported acquisition source, accounting for 200/400 participants (50.0%), followed by leftover medicines (60/400, 15.0%), online sources (47/400, 11.8%), family members or friends (37/400, 9.3%), previous prescriptions (36/400, 9.0%), and other sources (20/400, 5.0%). Acquisition-source distribution differed significantly according to self-medication status (*P* < 0.001) (Table 4).

**Table 4:** Primary reported antibiotic acquisition source according to self-medication status.

Primary reported acquisition source	Overall, n (%)	Self-medication, n/N (%)	Physician-prescribed, n/N (%)
**Pharmacy**	200 (50.0)	151/200 (75.5)	49/200 (24.5)
**Previous prescription**	36 (9.0)	15/36 (41.7)	21/36 (58.3)
**Family member or friend**	37 (9.3)	21/37 (56.8)	16/37 (43.2)
**Online source**	47 (11.8)	21/47 (44.7)	26/47 (55.3)
**Leftover medicine**	60 (15.0)	28/60 (46.7)	32/60 (53.3)
**Other**	20 (5.0)	8/20 (40.0)	12/20 (60.0)

Source categories were mutually exclusive. The overall association between acquisition source and self-medication status remained significant after accounting for the reconstructed cluster/stratum design (*P* < 0.001). Acquisition source describes how the antibiotic was obtained and is distinct from whether a physician was involved in prescribing the index course. Accordingly, a physician-prescribed course could coexist with leftover stock or a previous prescription as the acquisition source; all 400 records were audited against the original field records, and no additional discrepancies were identified.

### Reported indications and WHO AWaRe classification

Among the 244 participants who self-medicated, the most frequently reported indications were fever (203/244, 83.2%), diarrhea (183/244, 75.0%), common cold (175/244, 71.7%), cough (165/244, 67.6%), burning micturition (143/244, 58.6%), and sore throat (79/244, 32.4%). Participants could report more than one indication (Table 5).

**Table 5:** Reported indications, antibiotic-identity verification, and WHO AWaRe analyses among participants who self-medicated.

Measure	n/N (%)	Design-aware 95% CI
**Panel A. Reported indications**		
Fever	203/244 (83.2)	—
Diarrhea	183/244 (75.0)	—
Common cold	175/244 (71.7)	—
Cough	165/244 (67.6)	—
Burning micturition	143/244 (58.6)	—
Sore throat	79/244 (32.4)	—
**Panel B. Antibiotic-identity verification**		
Prescription reviewed	0/244 (0.0)	—
Antibiotic strip reviewed	70/244 (28.7)	—
Package/container reviewed	52/244 (21.3)	—
Photograph reviewed	30/244 (12.3)	—
Participant self-report only	49/244 (20.1)	—
Unknown/unclassifiable (verification not applicable)	43/244 (17.6)	—
Objectively verified total (levels 1-4)	152/244 (62.3)	—
Classifiable AWaRe total	201/244 (82.4)	—
**Panel C. WHO AWaRe distribution and sensitivity analyses**		
Access, among classifiable episodes	56/201 (27.9)	21.6-34.1
Watch, among classifiable episodes	137/201 (68.2)	61.6-74.7
Reserve, among classifiable episodes	8/201 (4.0)	0.9-7.1
Watch or Reserve, among classifiable episodes	145/201 (72.1)	65.9-78.4
Watch or Reserve, using all self-medication episodes	145/244 (59.4)	52.7-66.1
Lower-bound scenario: all unclassifiable episodes assigned to Access	145/244 (59.4)	52.7-66.1
Upper-bound scenario: all unclassifiable episodes assigned to Watch/Reserve	188/244 (77.0)	72.1-82.0
Watch or Reserve among objectively verified episodes	117/152 (77.0)	70.4-83.5

Participants could report more than one indication; indication percentages therefore do not sum to 100%. Verification refers to the highest available evidence supporting antibiotic identity for the self-medication episode. Levels 1-4 were considered objectively verified. WHO AWaRe classification used the 2025 WHO framework. The primary Access/Watch/Reserve distribution used 201 classifiable episodes; 43/244 (17.6%) episodes remained unclassifiable. Best-/worst-case bounds assign all 43 unclassifiable episodes to AWaRe, respectively. Design-aware CIs account for the reconstructed 50-PSU/four-stratum sampling design and are unweighted.

AWaRe, Access, Watch, and Reserve; CI, confidence interval; PSU, primary sampling unit; WHO, World Health Organization.

Antibiotic identity was objectively verified in 152/244 self-medication episodes (62.3%), while 49 (20.1%) were classifiable from participant report alone, and 43 (17.6%) remained unclassifiable. Among the 201 classifiable episodes, 56 (27.9%) involved Access antibiotics, 137 (68.2%) Watch antibiotics, and eight (4.0%) Reserve antibiotics. Watch or Reserve agents therefore accounted for 145/201 episodes (72.1%; design-aware 95% CI 65.9-78.4%).

Using all 244 self-medication episodes as the denominator, the observed Watch/Reserve proportion was 59.4% (95% CI 52.7-66.1%). Under extreme assumptions for the 43 unclassifiable episodes, the corresponding proportion ranged from 59.4% to 77.0%. Restriction to the 152 objectively verified episodes yielded a Watch/Reserve proportion of 77.0% (117/152; 95% CI 70.4-83.5%) (Table 5).

## Discussion

In this community-based study of adults with recent antibiotic use in Narayanganj Sadar, 61.0% of participants self-medicated for their index antibiotic course. This estimate should be interpreted as a 6-month proportion among recent antibiotic users, not as the prevalence of self-medication in the general adult population. After accounting for the reconstructed stratified cluster design, pharmacy access, poor antibiotic knowledge, lower educational attainment, and absence of AMR awareness remained associated with self-medication. The stewardship concern extended beyond unsupervised use itself: 72.1% of classifiable self-medicated antibiotics belonged to the WHO Watch or Reserve groups.

Direct comparison with previous Bangladesh estimates requires caution because study populations, recall periods, and definitions differ. A community survey in Rajshahi reported self-medication among 26.7% of participants [5], whereas pharmacy-based studies have documented substantial non-prescription antibiotic purchasing and dispensing [6,7]. Studies among university students and Indigenous communities have similarly identified self-medication as an important component of antibiotic use [9,10]. Our estimate is therefore best interpreted within its deliberately restricted denominator of adults who had recently used an antibiotic, rather than as evidence that self-medication is more common in Narayanganj than in these populations.

Pharmacy access showed the largest adjusted association with self-medication. This finding requires particularly careful interpretation. In the revised analysis, pharmacy access denotes pharmacy as the reported acquisition source for the index antibiotic course; it does not represent an independently measured pharmacy recommendation and does not establish that pharmacy personnel caused self-medication. Pharmacies may supply antibiotics prescribed by qualified clinicians as well as antibiotics obtained without physician involvement, and Bangladesh research has described drug shops as central nodes within multiple formal and informal pathways to medicines [13]. The observed association therefore identifies pharmacy access as an important feature of the medicine-acquisition environment rather than a causal determinant. It nevertheless suggests that community pharmacies are an important setting in which future stewardship strategies could be evaluated, alongside measures addressing access to qualified clinical assessment and patient demand. The present study did not test whether any pharmacy-centered intervention would reduce self-medication.

Poor antibiotic knowledge and lack of AMR awareness were also associated with self-medication, while lower educational attainment remained associated after adjustment for household income and other covariates. These findings are consistent with previous evidence linking antimicrobial knowledge and health literacy with self-medication behaviors [3,10]. However, the knowledge measure used here was a single bacterial-versus-viral recognition indicator rather than a validated multidimensional scale. Moreover, knowledge and AMR awareness were measured at interview, whereas the index antibiotic course occurred within the preceding 6 months. Their temporal ordering cannot therefore be established: lower knowledge may have preceded the self-medication decision, but the cross-sectional data cannot demonstrate this. These measures should consequently be interpreted as associated characteristics rather than causal antecedents.

The reported indications further illustrate the clinical uncertainty inherent in unsupervised antibiotic use. Fever, diarrhea, common cold, cough, and urinary symptoms were frequently reported, often in combination. Some such presentations may be viral or self-limiting, whereas others may represent bacterial infections for which antibiotic treatment is appropriate. Because the study did not clinically adjudicate individual episodes or incorporate diagnostic testing, it cannot determine whether a given antibiotic course was clinically indicated. The findings therefore characterize patterns of self-medication and antibiotic exposure, not the appropriateness of treatment for individual illnesses.

The AWaRe findings provide an additional stewardship signal. Among 201 self-medication episodes in which the antibiotic could be classified, 68.2% involved Watch agents and 4.0% Reserve agents, yielding a combined Watch/Reserve proportion of 72.1%. Because 43 episodes were unclassifiable, reliance on the classifiable denominator alone could overstate or understate the overall distribution. Sensitivity analyses therefore considered all 244 self-medication episodes: the Watch/Reserve proportion was 59.4% using the full denominator and ranged from 59.4% to 77.0% under extreme assumptions for unclassifiable episodes. Among the 152 episodes with objectively verified antibiotic identities, 77.0% involved Watch or Reserve agents. The consistency of these analyses indicates that the predominance of higher-stewardship-priority antibiotics was not confined to self-reported drug identities, although the study cannot infer population-level antibiotic consumption from these episode-based data. These episode-based AWaRe distributions should therefore be interpreted as a stewardship signal rather than as a measure of population-level antibiotic consumption [8].

Several features strengthen the study. Participants were recruited from 50 clusters spanning urban, semi-urban, peri-urban, and rural strata, and the revised analysis explicitly accounted for the recovered clustering and stratification rather than treating observations as independent. All antibiotic-specific variables were anchored to a common index course, reducing ambiguity when participants had used antibiotics more than once during the recall period. Prescription status and acquisition source were maintained as conceptually distinct variables and were audited against the original field records for all 400 participants. Antibiotic identities were verified where supporting evidence was available, and uncertainty in AWaRe classification was addressed through complementary denominator, bounding, and objectively verified sensitivity analyses. Modified Poisson regression provided prevalence ratios, which are more interpretable than odds ratios for a common outcome; the logistic sensitivity analysis produced directionally concordant findings.

The study also has important limitations. Its cross-sectional design precludes causal or temporal inference. Antibiotic use, physician involvement, symptoms, knowledge, and several other measures relied at least partly on participant recall and may be affected by recall, social desirability, or classification error despite procedures intended to reduce these biases. Antibiotic knowledge was assessed using a single operational item rather than a validated scale. In total, 17% of self-medication episodes could not be classified by AWaRe, although the sensitivity analyses make the resulting uncertainty explicit. Clinical records and diagnostic findings were unavailable, preventing assessment of treatment appropriateness. The original sample-size calculation did not incorporate a cluster design effect. Although cluster and stratum membership were recovered and incorporated into revised variance estimation, the original first-stage sampling-frame denominators and historical household counts required to reconstruct exact sampling weights were unavailable; analyses were therefore unweighted. Finally, Narayanganj Sadar was selected as a heterogeneous subnational setting rather than as a representative sample of Bangladesh, and extrapolation to other populations should be cautious.

## Conclusion

Antibiotic self-medication was common among recent antibiotic users in Narayanganj Sadar and was associated with pharmacy access, lower educational attainment, poor antibiotic knowledge, and lack of AMR awareness. Watch and Reserve antibiotics accounted for a substantial proportion of self-medicated agents, including in analyses restricted to objectively verified antibiotic identities. These findings highlight medicine-access pathways and antimicrobial literacy as relevant domains for stewardship, while prospective and intervention studies are needed to determine which strategies can reduce inappropriate non-prescription antibiotic use without impeding access to necessary treatment.

## Funding

This research did not receive any specific grant from funding agencies in the public, commercial, or not-for-profit sectors. Study costs were borne by the investigators, who had sole responsibility for the study design; data collection, analysis, and interpretation; manuscript preparation; and the decision to submit the manuscript for publication.

## Ethical approval

**Informed consent and patient details**: Written informed consent to take part in the study and to publish the article has been obtained from all participants or their legal representatives. The privacy rights of participants have been observed.

**Studies in human**: This study was performed in compliance with relevant laws, regulatory frameworks, and guidelines where the research took place.

This study was approved by the Department of Applied Health and Nutrition, RTM Al-Kabir Technical University.

(Approval No. 0992420007273006)

## Data sharing

Deidentified participant-level data and the data dictionary will be available from the corresponding author (syedrhode14@gmail.com) upon reasonable request for 5 years after publication. Access will require a methodologically sound proposal, investigator approval, and a signed data-use agreement.

## Declaration of generative AI and AI-assisted technologies in the manuscript preparation process

During the preparation of this work, the authors used ChatGPT (OpenAI) for language editing and manuscript formatting assistance. After using this tool, the authors reviewed and edited the content as needed and take full responsibility for the content of the published article.

## CRediT authorship contribution statement

**Syed Amir Al Shahid Rhode:** Writing – review & editing, Writing – original draft, Visualization, Validation, Supervision, Software, Project administration, Methodology, Formal analysis, Data curation, Conceptualization. **Tairin Sultana:** Writing – review & editing, Validation, Methodology. **Mahzabin:** Writing – review & editing, Validation. **Aaqib Adnan Likhon:** Writing – review & editing, Project administration, Investigation, Data curation. **Alif Ahnaf Shikhon:** Writing – review & editing, Project administration, Investigation. **Rifa Rafia:** Writing – review & editing, Project administration, Investigation.

## Declaration of competing interest

The authors have no competing interests to declare.

## Appendix Supplementary materials

Image, application 1

Image, application 2

Image, application 3

Image, application 4
