# Supplementary material for: Community antibiotic self-medication, pharmacy access, and WHO AWaRe classification of self-medicated antibiotics among adult antibiotic users in Narayanganj Sadar, Bangladesh: a multistage stratified cluster-sampled cross-sectional study
Source: IJID Reg. 2026 Sep 11;21:101009. doi: 10.1016/j.ijregi.2026.101009 (PMC13633893; doi:10.1016/j.ijregi.2026.101009)
Supplement: Supplementary file 1 [file mmc1.docx]

**Supplementary Methods**

**Contents**

[**SM1. Study design, setting, and stratification framework	2**](#_Toc237963822)

[**SM2. Cluster selection and within-cluster household sampling	4**](#_Toc237963823)

[**SM3. Eligibility, participant selection, and definition of the index antibiotic course	6**](#_Toc237963824)

[**SM4. Questionnaire development, translation, pilot testing, and field standardisation	7**](#_Toc237963825)

[**SM5. Operational definitions and decision algorithm	9**](#_Toc237963826)

[**SM6. Distinction between physician involvement and antibiotic acquisition source	14**](#_Toc237963827)

[**SM7. Pharmacy-access variable provenance and interpretation	16**](#_Toc237963828)

[**SM8. Antibiotic identity ascertainment and WHO AWaRe classification	18**](#_Toc237963829)

[**SM9. Household-size measurement and revised modelling	21**](#_Toc237963830)

[**SM10. Reconstruction of cluster and stratum identifiers and final design-aware analysis	22**](#_Toc237963831)

[**SM11. Measurement, temporal, and causal-interpretation constraints	27**](#_Toc237963832)

[**SM12. Post-review data audit, data integrity, and reproducibility safeguards	28**](#_Toc237963833)

# **SM1. Study design, setting, and stratification framework**

The study was a community-based cross-sectional survey conducted in Narayanganj Sadar, Bangladesh, from January to April 2026. The target population comprised adult residents with recent antibiotic exposure, defined as antibiotic use during the preceding six months. The sampling architecture was specified before field recruitment and was designed to ensure representation of communities with contrasting settlement patterns, economic activity, built environment, and access to urban services.

Narayanganj Sadar was stratified into four operational area types. These strata were not post hoc analytical categories; they were field-sampling categories used to organise cluster selection and recruitment. The operational definitions were as follows:

- Urban: areas within the core city characterised by continuous built-up development, high population density, and predominantly non-agricultural economic activity.
- Semi-urban: areas that were partially urbanised or situated immediately outside the core city, while retaining substantial urban characteristics and mixed land use.
- Peri-urban: transitional fringe areas in which urban and rural land uses coexisted and agricultural land was undergoing rapid conversion to urban use.
- Rural: areas in which agriculture or fishing remained the principal economic activity, built-up density was low, and urban services were largely absent.

The allocation of operational clusters was deliberately balanced across the four strata. Thirteen clusters were allocated to the urban stratum, 12 to semi-urban, 12 to peri-urban, and 13 to rural areas. Eight participants were recruited from each cluster, yielding the prespecified final sample of 400 participants.

**Supplementary Methods Table 1. Allocation of operational clusters and recruited participants by sampling stratum**

| **Sampling stratum** | **Operational clusters, n** | **Participants, n** | **Participants, %** |
| --- | --- | --- | --- |
| **Urban** | 13 | 104 | 26.0 |
| **Semi-urban** | 12 | 96 | 24.0 |
| **Peri-urban** | 12 | 96 | 24.0 |
| **Rural** | 13 | 104 | 26.0 |
| **Total** | **50** | **400** | **100.0** |

*The final stratum totals above reflect the reconstructed and verified cluster-to-stratum mapping used for the revised analysis. Each of the 50 clusters contributed exactly eight participants.*

# **SM2. Cluster selection and within-cluster household sampling**

**SM2.1 First-stage cluster selection**

Within each of the four predefined strata, operational clusters were selected by lottery. The resulting distribution was 13 urban, 12 semi-urban, 12 peri-urban, and 13 rural clusters. The field plan assigned two clusters to each of 25 trained data collectors, thereby covering all 50 clusters.

The original first-stage denominators (that is, the complete number of potentially selectable clusters in each stratum) were not retained in the analytical archive. Consequently, first-stage selection probabilities and design weights cannot be reconstructed reliably and no retrospective weighting scheme is imposed.

**SM2.2 Household enumeration and calculation of the sampling interval**

Household counts were obtained separately for each selected cluster before systematic household recruitment. Because the target was eight participants per cluster, a cluster-specific household sampling interval (k) was calculated as:

k = number of households in the cluster / 8

When the quotient was not an integer, k was rounded to the nearest whole number. Thus, household selection was based on a cluster-specific interval rather than a single interval applied across the entire study area.

**SM2.3 Random start and systematic household progression**

For each cluster, the starting household was selected by lottery from households numbered 1 through k. After the random start, the field team approached every kth household according to the calculated interval. If a selected household contained no eligible participant, the team advanced by a further k households rather than substituting the immediately adjacent household. The same rule was followed when participation could not be obtained from a selected household. This procedure preserved the prespecified systematic interval during field recruitment.

**SM2.4 Selection when more than one eligible adult was present**

When more than one eligible adult was present in a selected household, a single participant was chosen by lottery. This prevented interviewer discretion or household availability from determining which eligible adult entered the study.

**SM2.5 Field-team allocation**

Twenty-five data collectors conducted recruitment. Each was assigned two operational clusters. The cluster assignment structure served both as a field-management mechanism and as an audit trail for reconstructing participant-to-cluster membership during manuscript revision.

# **SM3. Eligibility, participant selection, and definition of the index antibiotic course**

Eligible participants were residents of Narayanganj Sadar aged 18 years or older who had lived in the study area for at least six months and had used an antibiotic during the preceding six months. Healthcare professionals and healthcare students were excluded because their professional knowledge and access to medicines could differ systematically from those of the general community. Individuals who declined participation were not enrolled.

The six-month eligibility window could encompass more than one episode of antibiotic use. The field protocol therefore specified that, when a participant had used antibiotics on more than one occasion during the recall period, all antibiotic-specific questions referred to the last antibiotic course. Prescription status, acquisition source, indication, antibiotic identity, and subsequent classification were therefore anchored to the same index course rather than to an arbitrarily selected or most memorable episode.

This distinction is important because the study estimated the proportion of self-medication among recent antibiotic users rather than the population prevalence of any self-medication event occurring at any point during the six-month period. Each participant contributed one index course to the primary analysis.

# **SM4. Questionnaire development, translation, pilot testing, and field standardisation**

**SM4.1 Development and expert review**

The study protocol and structured questionnaire were developed for the study and reviewed before field implementation by a four-member multidisciplinary group comprising two public-health specialists, one epidemiologist, and one infectious-disease specialist. The available protocol does not document adaptation from a named externally validated parent questionnaire; the instrument is therefore described as study-developed and expert reviewed rather than as an adaptation of a specific published scale.

The instrument is therefore described as expert reviewed and pilot tested rather than as formally psychometrically validated. No claim is made that the full questionnaire, or the single-item antibiotic-knowledge indicator derived from it, constituted a validated scale.

**SM4.2 Translation and language of administration**

The questionnaire was developed in English and translated into Bangla by a professor of Bangla. Field administration was conducted in Bangla. The materials reviewed document this English-to-Bangla translation; no additional translation procedure is asserted beyond what is recorded in the protocol.

**SM4.3 Pilot testing**

Before formal data collection, the questionnaire and field procedures were pilot tested among 40 participants. Pilot participants were not included in the final analytical sample of 400. The available protocol confirms the pilot sample and its exclusion from the final dataset; it does not preserve a separate formal report of pilot-performance metrics, and none are inferred retrospectively.

**SM4.4 Training and standardisation of data collectors**

All 25 data collectors underwent intensive training before field deployment. The archived materials confirm standardised pre-field training but do not retain a complete itemised training syllabus; accordingly, the revised Methods report the training as implemented without attributing unrecorded modules or competencies to the programme.

# **SM5. Operational definitions and decision algorithm**

The principal analytical variables were derived from prespecified questionnaire items. The most consequential distinction was between whether a physician was involved in the decision to use the antibiotic and the route through which the participant physically obtained the antibiotic. These were conceptually different dimensions and were recorded separately in the field instrument.

**SM5.1 Definition of a physician**

For this study, a physician was operationally defined as an MBBS or BDS doctor registered with the Bangladesh Medical and Dental Council (BMDC) and possessing a BMDC registration number. The prescription-status question was: “Was the antibiotic prescribed by a physician?” The definition was provided to standardise what counted as physician involvement and to avoid classifying advice from unregistered or non-physician medicine sellers as physician prescribing.

**SM5.2 Definition of self-medication**

Antibiotic self-medication was operationally defined as antibiotic consumption for the index course that did not involve a physician as defined above. Participants reporting physician involvement in prescribing the index course were classified as physician-prescribed; participants without such involvement were classified as self-medication. The two outcome categories were mutually exclusive.

The wording “without a current prescription” used in the submitted manuscript could be misread as requiring a newly issued physical prescription document. The field protocol was anchored more directly to physician involvement. In the revised description, “physician-prescribed” therefore refers to the clinical prescribing decision for the index course, whereas the acquisition-source variable describes how or where the medicine was physically obtained.

**SM5.3 Antibiotic acquisition source**

Participants were asked: “Where did you obtain the antibiotic?” The mutually exclusive response categories were pharmacy, previous prescription, family/friend, online, leftover medicine, and other (with specification). The source variable therefore described the route of acquisition or access and was not, by itself, used to determine whether the index course was physician prescribed.

**SM5.4 Pharmacy-related reason for self-medication**

A separate questionnaire section asked about reasons for self-medication, and “pharmacy recommendation” was among the response options. As detailed in SM7, review of the analytical coding showed that the pharmacy-derived columns did not preserve an independently analysable recommendation construct. The revised analysis therefore does not treat pharmacy recommendation as an independently measured exposure. The single retained construct is pharmacy access, defined by reporting a pharmacy as the acquisition source for the index antibiotic course.

**SM5.5 Antibiotic-identity verification hierarchy**

For self-medication episodes, the highest available level of evidence supporting antibiotic identity was recorded using a prespecified hierarchy: (1) physician prescription reviewed; (2) antibiotic strip reviewed; (3) package/container reviewed; (4) photograph reviewed; (5) participant self-report only; and (88) not applicable. Only the highest available level was entered for each episode. Levels 1–4 constitute objective documentary or physical verification; level 5 denotes identification based on participant report alone.

**Supplementary Methods Table 2. Core questionnaire items, operational definitions, and analytical interpretation**

| **Construct** | **Field wording / rule** | **Coding or categories** | **Analytical interpretation** |
| --- | --- | --- | --- |
| **Index antibiotic course** | If >1 antibiotic course occurred in the preceding 6 months, use the last course | One index course per participant | All antibiotic-specific variables refer to the same episode |
| **Physician** | BMDC-registered MBBS or BDS doctor with a BMDC registration number | Operational definition | Standardises physician involvement |
| **Prescription status** | “Was the antibiotic prescribed by a physician?” | Yes / No | Clinical prescribing involvement for the index course |
| **Self-medication** | Antibiotic consumption that did not involve a physician | Yes / No | Primary outcome; mutually exclusive with physician-prescribed use |
| **Acquisition source** | “Where did you obtain the antibiotic?” | Pharmacy; previous prescription; family/friend; online; leftover medicine; other | Route through which medicine was obtained; not the prescribing decision |
| **Pharmacy-related questionnaire item** | Reasons for self-medication included pharmacy recommendation | Recorded in field instrument; not independently preserved in analysis | No separate recommendation exposure; final construct is pharmacy access from acquisition source |
| **Identity verification** | Highest level of verification available | 1 prescription; 2 strip; 3 package/container; 4 photograph; 5 self-report only; 88 N/A | Levels 1–4 objectively verified; level 5 self-report only |
| **Poor antibiotic knowledge** | Single bacterial-versus-viral recognition item | Adequate / poor | Single-item indicator, not a validated knowledge scale |
| **AMR awareness** | Whether participant had heard of antibiotic resistance | Aware / not aware | Measured at interview; temporality relative to index course cannot be established |

**SM5.6 Decision algorithm for primary classification**

The operational decision sequence can be summarised as follows:

- Identify the participant’s last antibiotic course within the preceding six months.
- Ask whether that course was prescribed by a BMDC-registered MBBS/BDS physician.
- If physician involvement was present, classify the index course as physician-prescribed; if absent, classify it as self-medication.
- Record the acquisition source separately, irrespective of prescription status.
- For self-medication episodes, ascertain the antibiotic identity and record the highest available verification level.
- Where the antibiotic identity is sufficiently identifiable, classify the agent according to the WHO Access, Watch, and Reserve framework; retain unidentified episodes as unclassifiable rather than forcing assignment.

# **SM6. Distinction between physician involvement and antibiotic acquisition source**

The submitted source table contained two combinations that could appear contradictory if “source” were interpreted as synonymous with prescribing status: 32 physician-prescribed participants reported leftover medicine as the acquisition source, and 21 physician-prescribed participants reported a previous prescription as the acquisition source. These 53 reviewer-flagged records were individually traced to the original field information. The audit was then extended to all 400 participants by comparing prescription status and antibiotic acquisition source against the original field records.

The audit confirmed that the 53 apparently discordant combinations were genuine. In the 32 leftover-medicine records, the index course involved a physician, but the participant already had the relevant antibiotic remaining from an earlier course and used that stock rather than obtaining a newly dispensed supply. In the 21 previous-prescription records, the participant had a history of recurrent infection for which the same antibiotic had been prescribed repeatedly; a physician was involved in the index course, and the participant used an earlier prescription containing the same antibiotic when obtaining or purchasing the medicine. Thus, the source field described the immediate acquisition pathway, while the prescription-status field described physician involvement in the clinical decision for the index course.

No additional discrepancies were identified in the remaining records. The full 400-record audit therefore resulted in no further correction of prescription status or antibiotic acquisition source and no change to the primary self-medication classification. Reuse of leftovers or an old prescription without physician involvement would have met the operational definition of self-medication; however, that was not the circumstance documented for the 53 reviewer-flagged physician-prescribed records.

**Supplementary Methods Table 3. Full record-level audit of prescription status and antibiotic acquisition source**

| **Audit component** | **n** | **Field-record finding** | **Analytical consequence** |
| --- | --- | --- | --- |
| **Physician-prescribed + leftover medicine** | 32 | Index course involved a physician; participant used antibiotic stock remaining from a previous course. | No recoding |
| **Physician-prescribed + previous prescription** | 21 | Current physician involvement confirmed; recurrent infection with repeated prescribing of the same antibiotic; earlier prescription used during acquisition. | No recoding |
| **Reviewer-flagged combinations subtotal** | 53 | Prescription status and acquisition source represented distinct dimensions. | No outcome recoding on this basis |
| **Full prescription/source audit** | 400 | All participant records were compared with the original field records; no additional discrepancies were identified. | No additional correction or outcome recoding |

**SM7. Pharmacy-access variable provenance and interpretation**

The original questionnaire contained two conceptually distinct prompts: the acquisition-source question (“Where did you obtain the antibiotic?”), for which pharmacy was one response option, and a reasons-for-self-medication section, for which pharmacy recommendation was one response option. During post-review data-provenance checking, however, the analytical columns labelled Pharmacy_influence, Reason_pharmacy, and Pharmacy_recommendation_raw were found to be identical in all 400 participants to the indicator that the acquisition source was a pharmacy. They therefore did not constitute independent measurements of three separate pharmacy mechanisms.

Because the analytical data did not independently preserve recommendation, dispensing, and participant-selected pharmacy purchase, those mechanisms cannot be decomposed retrospectively without introducing unsupported assumptions. The revision therefore consolidates the pharmacy-related information into one construct: pharmacy access, defined as reporting a pharmacy as the acquisition source for the index antibiotic course. No separate “pharmacy recommendation” exposure is analysed or claimed.

Pharmacy access is contemporaneous with the medicine-access pathway and may overlap conceptually with pathways into non-prescription antibiotic use. It is therefore analysed as an associated access characteristic, not as an independent causal antecedent. The design-aware crude prevalence ratio for pharmacy access was 1.62 (95% CI 1.40–1.89; p < 0.001), and the adjusted prevalence ratio was 1.50 (95% CI 1.29–1.74; p < 0.001). These estimates describe association only; they do not establish that pharmacy access, pharmacy personnel, or pharmacy recommendation caused self-medication, and the study did not evaluate the effectiveness of pharmacy-centred interventions.

**Supplementary Methods Table 4. Cross-tabulation of pharmacy access and self-medication status in the audited dataset**

| **Pharmacy access** | **Overall, n (%)** | **Self-medication, n/N (%)** | **Physician-prescribed, n/N (%)** |
| --- | --- | --- | --- |
| **Yes** | 200 (50.0) | 151/200 (75.5) | 49/200 (24.5) |
| **No** | 200 (50.0) | 93/200 (46.5) | 107/200 (53.5) |
| **Total** | **400 (100.0)** | **244/400 (61.0)** | **156/400 (39.0)** |

*Interpretive note: pharmacy access was present in 151 of 244 participants who self-medicated and in 49 of 156 participants whose index course was physician-prescribed; conversely, 93 participants self-medicated without pharmacy access. Pharmacy access therefore shows substantial but incomplete overlap with the outcome and is not mathematically equivalent to self-medication. This contemporaneous overlap is explicitly treated as a measurement and interpretation limitation.*

# **SM8. Antibiotic identity ascertainment and WHO AWaRe classification**

**SM8.1 Verification hierarchy and analysis population**

The verification hierarchy was applied to self-medication episodes because antibiotic identity and WHO AWaRe classification were secondary analyses of the self-medication subset. Physician-prescribed episodes were not subjected to this self-medication verification workflow and were coded as not applicable for this variable.

Among the 244 self-medication episodes in the audited dataset, 152 (62.3%) had objective verification through an antibiotic strip, package/container, or photograph; 49 (20.1%) relied on participant self-report alone; and 43 (17.6%) were not classifiable by antibiotic identity and remained outside the classifiable AWaRe denominator. No self-medication episode was recorded at the highest hierarchy level of a physician prescription reviewed. The verification workflow was applied to self-medication episodes because physician-prescribed episodes were not part of the self-medication AWaRe analysis.

**SM8.2 AWaRe classification and handling of unidentified antibiotics**

Identifiable antibiotics used in self-medication were classified according to the 2025 WHO Access, Watch, and Reserve framework. Episodes for which the antibiotic could not be identified reliably were retained as unknown/unclassifiable and were not forced into an AWaRe category. Among 201 classifiable self-medication episodes, 56 were Access (27.9%; design-aware 95% CI 21.6%–34.1%), 137 were Watch (68.2%; 61.6%–74.7%), and 8 were Reserve (4.0%; 0.9%–7.1%).

Four complementary AWaRe estimands were evaluated in the final analysis: (1) the distribution among the 201 classifiable self-medication episodes; (2) the Watch/Reserve proportion using all 244 self-medication episodes as the denominator; (3) best- and worst-case bounds assigning all 43 unclassifiable episodes alternatively to Access or to Watch/Reserve; and (4) a sensitivity analysis restricted to objectively verified antibiotic identities (verification levels 1–4). All confidence intervals reported for these proportions use the recovered cluster/stratum design.

**Supplementary Methods Table 5. Antibiotic-identity verification and AWaRe analysis populations among self-medication episodes**

| **Verification / analysis category** | **n** | **% of 244 self-medication episodes** | **Analytical role** |
| --- | --- | --- | --- |
| **Prescription reviewed** | 0 | 0.0 | Highest verification level; none observed in self-medication subset |
| **Antibiotic strip reviewed** | 70 | 28.7 | Objectively verified |
| **Package/container reviewed** | 52 | 21.3 | Objectively verified |
| **Photograph reviewed** | 30 | 12.3 | Objectively verified |
| **Participant self-report only** | 49 | 20.1 | Classifiable if antibiotic name identifiable; sensitivity distinction from objective verification |
| **Unclassifiable / verification N/A** | 43 | 17.6 | Unknown identity; excluded from classifiable AWaRe denominator and included in uncertainty bounds |
| **Objectively verified total** | 152 | 62.3 | Verified-only AWaRe sensitivity population |
| **Classifiable AWaRe total** | 201 | 82.4 | Primary classifiable AWaRe distribution denominator |

**SM8.3 Sensitivity-analysis definitions**

Watch or Reserve antibiotics accounted for 145/201 classifiable self-medication episodes (72.1%; design-aware 95% CI 65.9%–78.4%). Using all self-medication episodes as the denominator, the corresponding observed proportion was 145/244 (59.4%; 52.7%–66.1%). Under the lower-bound scenario, all 43 unclassifiable episodes were assigned to Access, leaving Watch/Reserve at 145/244 (59.4%; 52.7%–66.1%). Under the upper-bound scenario, all 43 were assigned to Watch/Reserve, yielding 188/244 (77.0%; 72.1%–82.0%).

Within the objectively verified subset (n=152), the audited data comprised 35 Access, 109 Watch, and 8 Reserve episodes; 117/152 were therefore Watch/Reserve (77.0%; design-aware 95% CI 70.4%–83.5%). The verified-only analysis is treated as a sensitivity analysis rather than as a replacement for the classifiable-episode analysis, because excluding identifiable self-report-only episodes would alter the analysis population.

# **SM9. Household-size measurement and revised modelling**

Household size was recorded as the number of family/household members rather than as an eligibility criterion. In the audited dataset, household sizes ranged from two to seven members: 30 participants (7.5%) lived in two-member households, 72 (18.0%) in three-member households, 271 (67.8%) in four-member households, 13 (3.2%) in five-member households, 6 (1.5%) in six-member households, and 8 (2.0%) in seven-member households. No one-person household was present in the recruited sample; this reflects the realised sample rather than a protocol-level exclusion.

The submitted analysis dichotomised household size as <5 versus ≥5 members. For the revised primary regression, household size was modelled more granularly as 2, 3, 4, and ≥5 members, with two-member households as the reference category. Exact sizes 5, 6, and 7 were pooled as ≥5 because their individual counts were sparse (n=13, n=6, and n=8), and separate adjusted coefficients would therefore have been unnecessarily unstable. The adjusted prevalence ratios were 1.02 (95% CI 0.74–1.41; p = 0.882) for 3 versus 2 members, 1.14 (0.88–1.47; p = 0.303) for 4 versus 2, and 1.10 (0.78–1.54; p = 0.586) for ≥5 versus 2. The joint design-aware test showed no independent household-size association after adjustment (F(3,46) = 0.695; p = 0.560).

Values coded as 88 represent structural non-applicability rather than ordinary missing data. Such values are excluded only from analyses for which the underlying item was not applicable. No statistical imputation is used to replace structural non-applicability or unknown antibiotic identity.

# **SM10. Reconstruction of cluster and stratum identifiers and final design-aware analysis**

**SM10.1 Recovery of field-design identifiers**

Cluster and stratum information was used operationally during fieldwork but was not retained in the analytical master file used for the submitted manuscript. During manuscript revision, the original protocol, field-allocation records, data-collector assignments, and participant records were re-examined to reconstruct the sampling-design variables. All 50 operational clusters were recovered, each containing exactly eight participants, and each cluster was mapped to its original area-type stratum. The reconstructed participant distribution was 104 urban, 96 semi-urban, 96 peri-urban, and 104 rural participants.

This reconstruction permits the revised analysis to distinguish the clustering and stratification components of the sampling design rather than treating the 400 observations as statistically independent. The original statement that cluster and stratum identifiers were unavailable applies to the submitted analytical file; it does not imply that the field design itself lacked these identifiers.

**SM10.2 Sampling weights and finite-population information**

Two pieces of information required for reconstruction of exact design weights were not preserved: the complete first-stage sampling-frame denominators within each stratum and the exact historical household counts used to calculate k within each cluster. Because the overall inclusion probability for each participant cannot therefore be reconstructed with sufficient confidence, no post hoc sampling weights or finite-population corrections were manufactured. All revised analyses are explicitly unweighted, with an analysis weight of 1 for every participant.

**SM10.3 Final design-aware inferential framework**

The final analysis specified the 50 reconstructed operational clusters as primary sampling units and the four area types as sampling strata. Variance estimation used a stratified cluster (Taylor/survey sandwich) estimator in which primary-sampling-unit score contributions were centred within strata. Inference used 46 design degrees of freedom, calculated as 50 primary sampling units minus 4 strata, with t-based confidence intervals and two-sided tests. This replaces the submitted individual-level Huber–White variance estimator, which did not account for within-cluster correlation.

The primary association model was modified Poisson regression with a log link and the recovered stratified-cluster variance estimator. The adjusted model included age group, sex, marital status, educational attainment, monthly household income, household size modelled as 2/3/4/≥5 members, the single-item antibiotic-knowledge indicator, antimicrobial-resistance awareness, and pharmacy access. Effect estimates are prevalence ratios rather than risks. Multi-degree-of-freedom adjusted tests were used for multi-category covariates. Analyses were conducted using Python version 3.13.5, principally with pandas version 2.2.3, SciPy version 1.17.0, and statsmodels version 0.14.6. Stratified-cluster sandwich covariance matrices and t-based inference were calculated from primary-sampling-unit score contributions centred within strata.

The primary six-month proportion of self-medication among recruited recent antibiotic users was 244/400 (61.0%), with a design-aware 95% confidence interval of 55.6%–66.4%. The principal adjusted associations remained statistically significant after design-aware reanalysis: low education, aPR 1.30 (95% CI 1.09–1.55; p = 0.005); poor antibiotic knowledge, aPR 1.46 (1.24–1.73; p < 0.001); no antimicrobial-resistance awareness, aPR 1.22 (1.06–1.41; p = 0.008); and pharmacy access, aPR 1.50 (1.29–1.74; p < 0.001). Low household income was not statistically significant after adjustment (aPR 1.13, 0.97–1.32; p = 0.121). The 61.0% estimate is interpreted specifically as a six-month proportion among recent antibiotic users in the recruited sample, not as the prevalence of antibiotic self-medication in the general adult population of Narayanganj Sadar or Bangladesh.

**Supplementary Methods Table 6. Summary of post-review methodological reconstruction, audit findings, and analytical consequences**

| **Issue** | **Source used for reconciliation** | **Finding** | **Consequence for revised manuscript / analysis** |
| --- | --- | --- | --- |
| Cluster membership | Original protocol, field-allocation records, participant records | 50 clusters recovered; 8 participants in each cluster | Cluster specified as primary sampling unit in revised inference |
| Area-type stratum | Original protocol and cluster allocation | 13 urban, 12 semi-urban, 12 peri-urban, 13 rural clusters; participant totals 104/96/96/104 | Stratification incorporated; area distribution reported |
| Within-cluster household selection | Original protocol | Cluster-specific k; random start by lottery; every kth household; same interval maintained after ineligibility/non-response | Sampling procedure made reproducible |
| Index antibiotic episode | Original protocol | Last antibiotic course within 6-month recall period | Prescription, source, indication, and AWaRe refer to a common index episode |
| Full prescription/source audit | Original field records for all 400 participants | All 400 records audited; 53 reviewer-flagged combinations confirmed genuine; no other discrepancies found | No further correction or outcome recoding |
| Pharmacy-access coding | Questionnaire, data dictionary, audited analytical dataset | Pharmacy_influence, Reason_pharmacy, and Pharmacy_recommendation_raw were 400/400 identical to pharmacy acquisition source | Single pharmacy-access construct; no separate recommendation claim; non-causal interpretation |
| Identity verification | Original field hierarchy and analytical data | 152/244 (62.3%) self-medication episodes objectively verified; 49 self-report only; 43 unclassifiable | Verified-only and uncertainty-bound AWaRe analyses reported |
| Household size | Audited participant data and design-aware reanalysis | Observed sizes 2–7; revised regression uses 2/3/4/≥5; joint F(3,46) = 0.695, p = 0.560 | Granular specification; no independent adjusted association |
| Questionnaire development | Original protocol | Multidisciplinary expert review; Bangla translation; 40-person pilot; 25 trained collectors | Report as expert reviewed and pilot tested; do not claim formal validation |
| Sampling weights | Archived sampling information | First-stage denominators and exact historical cluster household counts not retained | Unweighted analysis (weight=1); no retrospective weights or FPC |
| Design-aware reanalysis | Reconstructed 50 PSUs and 4 strata | Taylor/survey sandwich variance; 46 design df; modified Poisson primary model; design-aware six-month proportion 61.0% (95% CI 55.6–66.4%) | Cluster/stratum-aware CIs and p-values replace submitted individual-level robust inference |

# **SM11. Measurement, temporal, and causal-interpretation constraints**

Several variables require deliberately non-causal interpretation. Pharmacy access is contemporaneous with the process through which the index antibiotic was obtained and overlaps conceptually with pathways into non-prescription use; it is therefore treated as an associated access characteristic rather than an independent causal antecedent. The study did not test whether pharmacy-centred interventions would reduce self-medication, and intervention effectiveness is not inferred from the observed association.

Antibiotic knowledge and awareness of antimicrobial resistance were measured at the time of interview, whereas the index antibiotic course occurred within the preceding six months. The temporal ordering of these measures relative to the antibiotic-use decision cannot be established. Associations involving knowledge or awareness therefore do not demonstrate that these characteristics preceded or caused the index self-medication episode.

“Poor antibiotic knowledge” is an operational single-item indicator based on recognition that antibiotics act against bacterial but not viral infections. It is not a validated multi-item knowledge scale, and construct validity should be interpreted accordingly. Similarly, the six-month recall period, interviewer-administered questionnaire, standardised definitions, and identity-verification procedures were intended to reduce recall and misclassification bias; they cannot be claimed to eliminate those biases.

The WHO AWaRe analysis is also subject to identification uncertainty. The primary classifiable distribution is supplemented by all-episode, best-/worst-case, and objectively verified-only analyses so that the effect of the 43 unclassifiable self-medication episodes is visible rather than hidden by denominator restriction.

# **SM12. Post-review data audit, data integrity, and reproducibility safeguards**

Each participant was assigned a unique study identifier, and the analytical dataset contained no direct personal identifiers. The originally submitted master dataset was retained for comparison during revision. Design-variable reconstruction and reanalysis were performed in separate working datasets, allowing revision-stage changes to be checked against the submitted data.

The post-submission methodological review distinguished three categories of change: (1) recovery of design information that existed during fieldwork but was omitted from the submitted analytical file (cluster and stratum identifiers); (2) clarification and audit of variable meaning without changing participant-level classification when the original field record supported the existing value (for example, physician involvement versus acquisition source); and (3) consolidation of analytical coding where multiple pharmacy-derived columns did not represent independent measurements. This distinction prevents methodological clarification from being confused with retrospective alteration of the original observations.

Prescription status and antibiotic acquisition source were audited against the original field records for all 400 participants. The 53 reviewer-flagged combinations were confirmed as genuine physician-directed index courses with either leftover stock or a previous prescription serving as the acquisition pathway, and no other prescription/source discrepancies were identified. No participant was reclassified merely because a source category appeared conceptually unusual. Unknown information remained unknown; sampling weights were not estimated from unverified historical denominators; and unclassifiable antibiotics were not assigned to an AWaRe category without sufficient identity information.

Together, these procedures provide an explicit chain from field protocol to questionnaire item, from questionnaire item to analytical variable, and from analytical variable to statistical interpretation. The purpose is not to imply a level of precision that the archived data cannot support, but to make the limits and strengths of the study design transparent and reproducible.

**Abbreviations**

*AMR, antimicrobial resistance; AWaRe, Access, Watch, Reserve; BDS, Bachelor of Dental Surgery; BMDC, Bangladesh Medical and Dental Council; MBBS, Bachelor of Medicine, Bachelor of Surgery; PSU, primary sampling unit; WHO, World Health Organization.*
