# Supplementary material for: Community antibiotic self-medication, pharmacy access, and WHO AWaRe classification of self-medicated antibiotics among adult antibiotic users in Narayanganj Sadar, Bangladesh: a multistage stratified cluster-sampled cross-sectional study
Source: IJID Reg. 2026 Sep 11;21:101009. doi: 10.1016/j.ijregi.2026.101009 (PMC13633893; doi:10.1016/j.ijregi.2026.101009)
Supplement: Supplementary file 2 [file mmc2.docx]

**Supplementary Table S1. Design-aware logistic regression sensitivity analysis of factors associated with antibiotic self-medication**

| **Predictor** | **Adjusted OR (95% CI)** | **p-value** |
| --- | --- | --- |
| **Age 30–39 vs 18–29 years** | 1.16 (0.54–2.50) | 0.694 |
| **Age 40–49 vs 18–29 years** | 1.12 (0.58–2.18) | 0.734 |
| **Age 50–59 vs 18–29 years** | 1.31 (0.59–2.94) | 0.501 |
| **Age ≥60 vs 18–29 years** | 1.26 (0.42–3.81) | 0.673 |
| **Female vs male** | 0.73 (0.42–1.24) | 0.233 |
| **Unmarried vs married** | 1.42 (0.78–2.58) | 0.239 |
| **Widowed vs married** | 1.27 (0.64–2.52) | 0.478 |
| **Divorced vs married** | 1.01 (0.48–2.14) | 0.975 |
| **Low education vs graduate/postgraduate** | 2.37 (1.37–4.10) | 0.003 |
| **Monthly income <30,000 vs ≥30,000 BDT** | 1.57 (0.87–2.82) | 0.131 |
| **Household size 3 vs 2** | 1.16 (0.54–2.51) | 0.701 |
| **Household size 4 vs 2** | 1.43 (0.78–2.61) | 0.245 |
| **Household size ≥5 vs 2** | 1.22 (0.48–3.08) | 0.674 |
| **Poor antibiotic knowledge** | 3.24 (2.00–5.25) | <0.001 |
| **No antimicrobial-resistance awareness** | 2.00 (1.20–3.33) | 0.009 |
| **Pharmacy access** | 3.34 (2.16–5.18) | <0.001 |

*OR, odds ratio; CI, confidence interval; BDT, Bangladeshi taka. This model is a sensitivity analysis only. Variance estimation was unweighted and design-aware, specifying the same 50 reconstructed primary sampling units and four sampling strata as the primary modified Poisson analysis. Reference groups and covariate specification were identical to the primary model, including household size coded as 2 (reference), 3, 4, and ≥5. Because antibiotic self-medication was common, odds ratios are expected to be farther from the null than corresponding prevalence ratios; modified Poisson prevalence ratios remain the primary estimands.*
