# Supplementary material for: Community antibiotic self-medication, pharmacy access, and WHO AWaRe classification of self-medicated antibiotics among adult antibiotic users in Narayanganj Sadar, Bangladesh: a multistage stratified cluster-sampled cross-sectional study
Source: IJID Reg. 2026 Sep 11;21:101009. doi: 10.1016/j.ijregi.2026.101009 (PMC13633893; doi:10.1016/j.ijregi.2026.101009)
Supplement: Supplementary file 3 [file mmc3.docx]

**Supplementary Table S2. Concordance of principal adjusted associations between the primary modified Poisson model and logistic sensitivity analysis**

| **Predictor** | **Adjusted PR (95% CI)** | **PR p-value** | **Adjusted OR (95% CI)** | **OR p-value** | **Direction consistent** |
| --- | --- | --- | --- | --- | --- |
| **Low education vs graduate/postgraduate** | 1.30 (1.09–1.55) | 0.005 | 2.37 (1.37–4.10) | 0.003 | Yes |
| **Monthly income <30,000 vs ≥30,000 BDT** | 1.13 (0.97–1.32) | 0.121 | 1.57 (0.87–2.82) | 0.131 | Yes |
| **Poor antibiotic knowledge** | 1.46 (1.24–1.73) | <0.001 | 3.24 (2.00–5.25) | <0.001 | Yes |
| **No antimicrobial-resistance awareness** | 1.22 (1.06–1.41) | 0.008 | 2.00 (1.20–3.33) | 0.009 | Yes |
| **Pharmacy access** | 1.50 (1.29–1.74) | <0.001 | 3.34 (2.16–5.18) | <0.001 | Yes |

*PR, prevalence ratio; OR, odds ratio; CI, confidence interval; BDT, Bangladeshi taka. Both models used the same adjusted covariate set and the reconstructed unweighted stratified-cluster design. The logistic model is presented only to assess consistency of direction and approximate strength. Its odds ratios should not be interpreted as prevalence ratios because the outcome is common. The primary inferential results are those from the modified Poisson model.*
