# Supplementary material for: Community antibiotic self-medication, pharmacy access, and WHO AWaRe classification of self-medicated antibiotics among adult antibiotic users in Narayanganj Sadar, Bangladesh: a multistage stratified cluster-sampled cross-sectional study
Source: IJID Reg. 2026 Sep 11;21:101009. doi: 10.1016/j.ijregi.2026.101009 (PMC13633893; doi:10.1016/j.ijregi.2026.101009)
Supplement: Supplementary file 4 [file mmc4.docx]

**Community antibiotic self-medication, pharmacy access, and WHO AWaRe classification of self-medicated antibiotics among adult antibiotic users in Narayanganj Sadar, Bangladesh: a multistage stratified cluster-sampled cross-sectional study**

**STROBE Checklist**

| **Item** | **Topic** | **STROBE recommendation (condensed)** | **Where reported** |
| --- | --- | --- | --- |
| **1(a)** | Title and abstract | Identify the study design in the title or abstract. | Title, lines 2-4; Abstract-Methods, lines 37-43. |
| **1(b)** | Title and abstract | Provide an informative, balanced summary of methods and findings. | Abstract-Objectives, Methods, Results, and Conclusions, lines 34-53. |
| **2** | Background/rationale | Explain the scientific background and rationale. | Introduction, lines 57-94. |
| **3** | Objectives | State specific objectives and prespecified hypotheses. | Introduction, lines 95-100. |
| **4** | Study design | Present key elements of the design early. | Title, lines 2-4; Methods-Study design and participants, lines 102-108. |
| **5** | Setting | Describe setting, locations, and relevant dates. | Introduction (setting rationale), lines 91-95; Methods, lines 103-108; Supplementary Methods SM1, lines 507-528. |
| **6(a)** | Participants | Give eligibility criteria and participant/outlet selection methods. | Methods, lines 103-125; Supplementary Methods SM2-SM3, lines 534-582. |
| **6(b)** | Participants | For matched studies, state matching criteria and numbers. | Not applicable: unmatched cross-sectional study (design reported at lines 102-108). |
| **7** | Variables | Define outcomes, exposures, predictors, confounders, and diagnostic criteria. | Methods-Outcome and study variables/AWaRe classification, lines 109-153; Supplementary Methods SM5 and SM7-SM9, lines 612-669 and 696-786. |
| **8** | Data sources/measurement | Describe data sources and measurement methods for each variable. | Methods-Sampling/data collection and measurement, lines 113-153; Supplementary Methods SM4-SM5 and SM8, lines 584-669 and 727-767. |
| **9** | Bias | Describe efforts to address potential sources of bias. | Methods, lines 109-125 and 145-153; Discussion, lines 295-319; Supplementary Methods SM11, lines 838-859. |
| **10** | Study size | Explain how the study size was determined. | Methods-Sampling and data collection, lines 119-121. |
| **11** | Quantitative variables | Explain handling of quantitative variables and groupings. | Methods, lines 138-143 and 164-166; Table 3 note, lines 435-445; Supplementary Methods SM9, lines 768-786. |
| **12(a)** | Statistical methods | Describe statistical methods, including control of confounding. | Methods-Statistical analysis, lines 154-170; Table 3 and note, lines 433-445; Supplementary Methods SM10.3, lines 808-833. |
| **12(b)** | Statistical methods | Describe subgroup and interaction analyses. | Not applicable: no subgroup or interaction analyses were prespecified or performed. |
| **12(c)** | Statistical methods | Explain how missing data were addressed. | Results, lines 179-187 (all 400 participants analysed); Supplementary Methods SM9, lines 784-786 (structural non-applicability/unknown identity; no imputation). |
| **12(d)** | Statistical methods | If applicable, describe sampling strategy in the analysis. | Methods, lines 113-121 and 154-167; Supplementary Methods SM10, lines 788-833. |
| **12(e)** | Statistical methods | Describe sensitivity analyses. | Methods, lines 149-153 and 168-170; Results, lines 210-212 and 229-233; Supplementary Tables S1-S2, lines 470-487; Supplementary Methods SM8.3, lines 755-766. |
| **13(a)** | Participants | Report numbers at each stage of the study. | Methods, lines 114-121; Results, lines 179-187 (400 enrolled and all analysed; 50 clusters/four strata). |
| **13(b)** | Participants | Give reasons for non-participation or exclusion. | Eligibility/exclusion criteria, lines 103-106; Results, lines 179-180 (screening, ineligibility, and refusal counts were not retained). |
| **13(c)** | Participants | Consider a flow diagram. | No flow diagram; unavailable screening/refusal counts are disclosed at lines 179-180. |
| **14(a)** | Descriptive data | Describe participant/outlet and exposure characteristics. | Results, lines 179-197 and 213-233; Tables 1, 2, 4, and 5. |
| **14(b)** | Descriptive data | Indicate missing data for each variable of interest. | Results, lines 179-187; Supplementary Methods SM9, lines 784-786. |
| **14(c)** | Descriptive data | Summarise follow-up time where applicable. | Not applicable: cross-sectional study without follow-up (lines 102-108). |
| **15** | Outcome data | Report numbers of outcome events or summary measures. | Results, lines 185-189 and 220-233; Tables 1 and 5. |
| **16(a)** | Main results | Give unadjusted and adjusted estimates with precision; state adjusted variables. | Methods, lines 162-170; Results, lines 199-212; Table 3 and note, lines 433-445. |
| **16(b)** | Main results | Report category boundaries when continuous variables are categorised. | Methods, lines 164-166; Table 3 and note, lines 433-445; Supplementary Methods SM9, lines 768-783. |
| **16(c)** | Main results | Translate relative risks into absolute risks when relevant. | Results, lines 191-197; Table 2 (absolute outcome proportions by category); Table 3 (relative estimates). |
| **17** | Other analyses | Report subgroup, interaction, and sensitivity analyses. | Logistic sensitivity analysis: Results, lines 210-212; Supplementary Tables S1-S2, lines 470-487. AWaRe sensitivity analyses: Results, lines 229-233; Table 5; Supplementary Methods SM8.3, lines 755-766. No subgroup/interaction analyses. |
| **18** | Key results | Summarise key results with reference to objectives. | Discussion, lines 235-242. |
| **19** | Limitations | Discuss limitations, direction, and magnitude of potential bias. | Discussion-Limitations, lines 306-319; Supplementary Methods SM11, lines 838-859. |
| **20** | Interpretation | Provide a cautious overall interpretation considering objectives and evidence. | Discussion, lines 235-294; Conclusion, lines 320-328. |
| **21** | Generalisability | Discuss external validity and generalisability. | Introduction, lines 91-95; Methods, lines 103-108; Discussion, lines 317-319; Conclusion, lines 320-328. |
| **22** | Funding | State funding source and role of funders. | Role of the funding source, lines 352-356. |
